# Playing Ludomotor Activities in Lleida During the Spanish Civil War: An Ethnomotor Approach

**DOI:** 10.3389/fpsyg.2020.612623

**Published:** 2021-01-12

**Authors:** Enric Ormo-Ribes, Pere Lavega-Burgués, Rosa Rodríguez-Arregi, Rafael Luchoro-Parrilla, Aaron Rillo-Albert, Miguel Pic

**Affiliations:** ^1^Motor Action Research Group (GIAM), INDEST, National Institute of Physical Education of Catalonia (INEFC), University of Lleida, Lleida, Spain; ^2^Motor Action Research Group (GIAM), INDEST, National Institute of Physical Education of Catalonia (INEFC), University of Barcelona, Barcelona, Spain; ^3^Motor Action Research Group (GIAM), Institute of Sport, Tourism, and Service, South Ural State University, Chelyabinsk, Russia

**Keywords:** ethnomotricity, motor praxeology, motor action science, traditional games, intangible cultural heritage, cluster

## Abstract

The traditional ludomotor activities (LA) are recognized by UNESCO as an intangible piece of cultural heritage. The ethnomotricity analyzes LA in its sociocultural context, taking into account the proprieties of rules or motor conditions (internal logic) and the link with local culture (external logic). The aim of this research was to identify and reveal the distinctive ethnomotor features of LA in order to understand the adaptations that occurred in the social scenario of the Spanish Civil War (1936–1939) in Lleida. The corpus of the research was constituted by 101 LA which were collected from the analysis of 20 semi-structured interviews. An “*ad hoc*” tool was designed and agreed upon by expert observers. It was comprised of a total of 27 ethnomotor variables related to LA. The experts achieved high reliability [Cohen’s kappa coefficient (κ) and Spearman’s correlation coefficient = 1] when the classification of LA was carried out on two different occasions. Descriptive statistics, cross-tabulations (Pearson’s chi-squared) effect sizes, and two-step clusters were performed by external and internal logic variables. The presence or absence of motor interaction (*X*^2^ = 9.029; *df* = 1; *p* < 0.003; *ES* = 0.298) was enlightening when comparing LA with and without a war connotation. On the other hand, the hierarchy of variables rested primarily on IL-Domain (Psycho-Coop-Oppo-Coop/Oppo) (PI = 1). Among other singularities, while two-step cluster analysis revealed a corresponding ethnomotor silhouette with cluster 1, with the warlike connotation (*n* = 48; 96.0%), its homologous structure was expressed (Cluster 2) in the absence of the warlike character (*n* = 26; 50%).

## Introduction

Different international organizations (UNESCO, 2003) and researchers (Lavega, 1995; Etxebeste, 2001; Lavega et al., 2006; Etxebeste et al., 2015; Parlebas, 2016; Lavega-Burgués and Navarro-Adelantado, 2019) have clearly shown that Traditional Sporting Games (TSG) are a piece of intangible cultural heritage (ICH).

According to Mauss (1996), each community has its own body of techniques; that is to say, symbolic creations close to the norms and values of that society. Through TSG, people use techniques that are specific to their community (Warnier, 2001; Parlebas, 2005; Rauber et al., 2014).

Motor praxeology provides scientific criteria to study TSG as ICH. TSG are motor situations whose rules are agreed by players themselves, from which a wide range of different playing styles emerge (Elloumi and Parlebas, 2009; Navarro-Adelantado and Lavega, 2017). Ludomotor activities without rules are called quasi-games (QTSG). These activities can vary continuously according to the choice of the players (Etxebeste et al., 2015). Both types of ludomotor activities (LA), TSG and QTSG, contain their own characteristics which can be scientifically revealed.

Motor praxeology establishes that each LA (TSG or QTSG) has an internal logic that determines a particular way of relating to other participants, to space, to time, and to material (Parlebas, 2020). Each one of these internal relationships originates from singular features, for example, relationship with others [motor interaction type: without interaction between participants (psychomotor)]; with motor interaction between players (sociomotor); domains of motor action (cooperation, opposition, or cooperation-opposition); relationship with space (stable or unstable space implying contingencies; space as q goal to be reached, fixed or in-motion, human or artificial); relationship with the material (with or without objects); and relationship with time (with or without a final outcome). Therefore, LA with a different internal logic (Lavega-Burgués et al., 2020) (e.g., tug of war, skittles, wrestling, hopscotch, or QTSG with boules) trigger different internal relationships that will lead to unequal motor conducts in the players. These motor conducts are loaded with signs, messages, and values promoted by their local community or culture (e.g., dominance relationships, gender roles, aggression, and permissible violence) (Berthoud-Aghilin, 1986; Etxebeste, 2001). As Warnier (2002) indicates, LA are effective body techniques of subjectification by which players become subjects who learn to govern themselves. According to Foucault, the power relationships are clues to understand how the body and the concept of subjectivity are related. Power does not have the same meaning in different societies. It is shaped by techniques of the self. In different situated interactions in ludomotor activities, each subject (not the individual or the actor) has the power to choose his or her own actions in a set of networks of interactions. In these playful contexts, the subject is “shaped, subjectified by its embodied material culture” (Warnier, 2011).

From this point of view, it is of great interest to apply the concept of ethnomotricity to the study of LA, understood as “*field and nature of the motor practices, considered from the point of view of their relation with the culture and the social environment in which they have been developed*” (Parlebas, 2001, p. 227).

An ethnomotor perspective provides tools to examine the particular features that may be contained in the rules or motor conditions of practice (internal logic) and the influences of the context (external logic) of LA.

In each context, the socio-cultural conditions of LA can be different in terms of protagonists (age, gender), zones (location: interior or exterior; setup: with or without preparation of the space), material (elaboration of the objects), and time (calendar). It is because of this that there is a need to contextualize any LA study in a specific historical period and geographical space.

In this research, we focus on the ethnomotor study of LA in the Catalan area of Lleida (Spain) during the last Spanish civil war (1936–1939). In this geographical area, the two opposing armies occupied strategic positions, simulating a chess game, with the alternation of advances, retreats, and maintenance of positions. In the area of Lleida, the positions of the two sides in dispute were maintained for months on both sides of the river that divides the city. The civilian population did not flee, living together and interacting with the military in many areas, and bearing witness to and engaging in scenarios of struggle where there were remains of war material (Mir, 1989).

In wartime contexts, the youngest gain very rapid access to the adult world and to new realities (Stargardt, 2006; Riot et al., 2014). The ludomotor activities (LA) with rules (TSG) or without rules (QTSG) of children and young people show that, instead of being considered simple “objects” of history, passive and anonymous victims of traumatic events, they should be seen as unique and active “subjects” of their own experiences (Stargardt, 2006).

During armed conflict, society becomes fragmented and structural violence may become a part of everyday social life (Galtung, 1996). The habits, ways of life, and need for survival affect the warlike LA of the youngest, incorporating singularities both in the features of the internal logic (motor relationships, way of starting and finishing the game, use of materials, and use of space) and in the socio-cultural conditions (zones, times, materials, and age and gender of the actors) (Ormo, 2017).

According to the ethnomotricity notion, several studies have been conducted from different contexts (e.g., Etxebeste, 2001; Lavega et al., 2006; Elloumi and Parlebas, 2009; Etxebeste et al., 2015; Navarro-Adelantado and Lavega, 2017; Lavega-Burgués and Navarro-Adelantado, 2019). Researchers have investigated, for instance, the predominance of games (TSG) over quasi-games (QTSG), the favoring of sociomotor games, the balance of games with or without a final score (outcome), the presence of TSG played by male, female, and mixed players, and the use of a wide range of objects. However, the ethnomotricity approach is yet to be examined in a war or conflict period.

We hypothesized that the war context had a direct influence on the body of techniques of those involved. This includes LA. Thus, it is possible to find different ethnomotor regularities in the rules and socio-cultural conditions of these LA with respect to periods without military conflict. In addition, typical elements of war (war material) could inspire new games or activities. These body of techniques would trigger motor conducts that would help build a subjectification linked to more or less imaginary combat situations (Warnier, 2001, 2002).

Based on this theoretical framework, this research proposed two objectives:

(a) To identify the distinctive ethnomotor features of the LA (with or without rules) played in Lleida during the Spanish civil war (1936–1939).
(b) To reveal the ethnomotor features of the LA (with or without rules) with and without war connotations in Lleida during the Spanish civil war related to the process of subjectification.

## Method

### Participants

This research had a corpus of 101 LA, identified through an oral source. The 20 interviewed people (men = 14; women = 6; Age Range; 69–78 years; M_age_ = 75.95, SD = 5.74) were identified with the help of city council social services in eight municipalities around the Province of Lleida. The inclusion criteria for participants were: (a) born between 1921 and 1930, (b) LA playing experience with war material, (c) adequate memory and mental skills, and (d) representing both genders.

The sample size was established by information saturation; the LA were cited by at least two different informants. The interviewees participated voluntarily and gave informed consent; the research was approved by the Ethics Committee for Clinical Research of the Catalan Sports Council 07/2019/CEICEGC.

### Instruments and Procedure

Twenty semi-structured interviews (Miles and Huberman, 1984; Mathew et al., 2019) were conducted and recorded individually (Sony Pressman M529V recorders). They were later transcribed into a Microsoft Word document. For each of the LA identified by the informants, the three phases established by Miles and Huberman (1984) were followed: (a) identification and exploration of LA. For each LA, the rules or motor conditions (internal logic) and the socio-cultural conditions of practice (external logic) were described according to the concept of ethnomotricity (Parlebas, 2001); (b) categorization in an Excel database of the 27 variables, of the internal logic referring to the relationship with others, with space, with the material, and with time, and of the external logic referring to protagonists, materials, zones, and moments of the game. In this study we used the same ethnomotor variables that have been explained in other methodological manuscripts (e.g., Lavega et al., 2006; Etxebeste et al., 2015). These variables are also used in other ethnomotricity research (e.g., Etxebeste, 2001; Navarro-Adelantado and Lavega, 2017; Lavega-Burgués and Navarro-Adelantado, 2019).

(c) interpretation of the results from an ethnomotor perspective.

A tool for the classification of LAs was designed “*ad hoc*” within the GIAM research group, with at least 10 researchers directly involved. After using the registration tool, it was implemented with modifications to ensure the quality of subsequent registrations. Previously applied procedures from the observational methodology were followed (Anguera et al., 2011; Arana et al., 2016; Lavega-Burgués et al., 2020). The expert process of classification of intra-observer TSGs was independently coded by two experts (twice). This action reached values of 1 by Cohen’s kappa coefficient (κ) and Spearman’s correlation coefficient, and ensured a very good data quality (0.81–1.00) (Landis and Koch, 1977).

#### Data Analysis

Due to the limitation of this article, the ethnomotor variables and their categories can be consulted in^1^.

Internal logic (IL) variables:

• Rules: (a) IL-Rule (Quasi-TSG or TSG);
• Relationship with others: (a) IL-Interaction (Psychomotor or Sociomotor); (b) IL-Domain (Psychomotor, Cooperation, Opposition, or Cooperation-Opposition); (c) IL-Psychomotor-Structure (Structure); (d) IL-Opposition-Structure (Structure); (e) IL-Coop/Oppo Structure (Structure); (f) IL-Communication-Exclusiveness (Exclusive Net or Ambivalent Net); (g) IL-Communication Stability (Stable Net or Unstable Net); (h) IL-Scoring-Interaction-Network (Cooperation Goal Net, Antagonistic Scoring Interactions Network, Mixed Scoring Interactions Network, or No Scoring Interactions Network); (i) IL-Opposition Type Interaction (Oppo-Body Contact or Oppo-Objects Contact); (j) IL-Opposition Body Aggression (Oppo-Body-Contact, Oppo-Body-Contact-Objects, Oppo-Strong Hit to Body, or Oppo Permanent Body Contact); and (k) IL-Roles-Changes-Network (Fixed Roles, Local Change Roles, or General Change Roles);
• Relationship with the material: (a) IL-Object (With objects or No objects);
• Relationship with the space: (a) IL-Space Stability (Stable Space or Unstable Space); (b) IL-Space-as-Objective (Fixed Human Space, Mobile Human Space, Fixed Artificial Space, or Mobile Artificial Space); (c) IL-Coop-Opposition-Charge-Distance (Almost Null Charge Distance, Reduced Contact Charge Distance, Median Contact Charge Distance, Long Charge Distance, or No Charge Distance); and (d) IL-Oppos-Guard-Distance (Almost Null Guard Distance, Reduce Guard Distance, Median Guard Distance, Long Guard Distance, or No Guard Distance);
• Relationship with time: (a) IL-Score;

External logic variables (EL):

• Military LA type: (a) EL-Warlike Ludomotor Activity (Warlike LA or non-Warlike LA); Warlike LA were identified as any LA that used some sort of object for war purposes (bomb, grenade, ammunition, etc.).
• Participants: (a) EL-Age (Children, Youth, Adults, and All Ages); (b) EL- Gender (Only Male, Only Female, Separated Male or Female, or Mixed Male-Female);
• Material: (a) EL-Material Provenance (Artificial Material or Natural Material); (b) EL-Material Modification (Modified Non-Military Material, Unmodified Military Material, Unmodified Military Material, or Without Material); and (c) EL-Material Elaboration (Handmade Material, External Made Material, or Without Material Elaboration);
• Zones: (a) EL-Zones Location (Inside-Zones, Outside-Zones, or In-Out-Zones); (b) EL-Zones Preparation (Prepared-Zone, Poorly Prepared-Zone, or Not Prepared-Zone);
• Moments: (a) EL-Calendar (With Calendar or Without Calendar).

All data were analyzed using the Statistical Package of Social Sciences (SPSS, version 25, IBM Corp., Armonk, NY, United States; IBM Corp. (2017)). In this research we used: (i) A comparative description of variables (these variables were individually considered); (ii) Crosstabulations [Pearson’s chi-squared test; (Gómez et al., 2015)], as well as adjusted residuals (*AR* > 1.96 or <−1.96) were used to know the association of each variable, in pairs, with respect to its warlike or non-warlike character; (iii) effect sizes [Cramer’s V test; (Volker, 2006)] were also applied. The strength of the interpretation was in accordance (Cohen, 1988) with 0.10 = small effect, 0.30 = medium effect, and 0.50 = large effect; and (iv) a classification technique belonging to the two-step cluster analysis family was applied (Fernández-Navarro et al., 2020), using log-likelihood selection (Burnham and Anderson, 2004) and Schwarz’s Bayesian Criterion (Clement, 2014) to reveal the predictive size and grouping of variables.

## Results

The games studied (*n* = 101) would require motor skills for their ludic development.

### Ethnomotor Features of the LA as a Whole

LA had a similar percentage among games (practices with rules) (51%) and quasi-games (50%). The 49% were activities with warlike connotations.

According to the internal logic, the following features were observed:

In relation to others: the psychomotor LA were slightly higher (54.9%) than the sociomotor LA (45.1%). In both groups of TSGs, the activities offered all possible interaction structures: Psychomotor LA (alone 28.4%), alternating comotricity 15.7% and simultaneous comotricity 10.8%, Sociomotor TSG: cooperative 17.6%; opposition 15.7% and cooperation-opposition 11.8%.

LA resulted in low rates of motor aggression, since most opposition (87.3%) and cooperation-opposition games (95.1%) were played without body contact with the opponents.

Regarding the material, a predominance (93.1%) of LA with objects was observed.

Regarding space, the majority (95.1%) of the LA were performed in a stable space.

Regarding to time, no LA had an accounting system.

From the external logic, the following features were observed:

The LA were mainly played by children (34.3%), by children and young people (37.3%), or by mixed ages (25.5%). Youth-only games accounted for 2.9%. At the same time, the LA were mainly male (54%) or mixed (41.2%). Only 2.9% were female-only and 1% were played by both genders separately.

Most of the LA (90.2%) were made with objects from a nearby artificial environment, 48% of which were military objects.

Most of the LA (71.8%) were played in outdoor spaces and in areas not specifically prepared for playing (86.3%). In addition, all the LA were carried out without a set schedule.

### Comparison of the Distinctive Ethnomotor Features of LA With and Without War Connotations

The data related to statistical analyses in order to compare LA with and without war connotations were presented in the following Table 1.

**TABLE 1:** Results cross-tables taking into account internal and external logic variables and LA with and without war features.

			LA with and without war feature			
			Warlike LA		Non-Warlike LA)	
Logic	Variables	Categories	*n*	*ar*	*N*	*ar*
Internal	ILrule	Play	35	4.2	15	–4.2
logic (IL)		Game	15	–4.2	37	4.2
	ILMinterac	Psycho	35	3	21	–3
		Socio	15	–3	31	3
	ILdomain	Coop	13	2.2	5	–2.2
		Coop-opo	2	–2.4	10	2.4
		Oppo	0	–4.3	16	4.3
		Psycho	35	3	21	–3
	ILobject	Obj	50	2.7	45	–2.7
		No obj	0	–2.7	7	2.7
	ILNetstab	Stab	50	2.5	46	–2.5
		Unstab	0	–2.5	6	2.5
	ILScore	FinTask	12	–0.8	16	0.8
		LimScore	0	–1.7	3	1.7
		LimTime	0	–1	1	1
		Wacco	38	1.6	32	–1.6
External	Elage	All	20	3.3	6	–3.3
logic (EL)		Child	0	–7.2	35	7.2
		Child-Youth	30	4.7	8	–4.7
		Youth	0	–1.7	3	1.7
	Elgender	Fem	0	–1.7	3	1.7
		Mas	41	5.4	15	–5.4
		Mix	8	–5.1	34	5.1
	ELMatprov	0_Obj	0	–2.7	7	2.7
		Art_Nat_obj	0	–1	1	1
		Art_Obj	50	3.3	42	–3.3
		Nat_Obj	0	–1.4	2	1.4
	Elzones	0-Obj	45	1.1	43	–1.1
		Low-Prep-Obj	1	–1.9	6	1.9
		Prep_Obj	4	4	3	–4

### Presence of Rules in LA With or Without a Warlike Character

There were significant differences (*X*^2^ = 17.275; *df* = 1; *p* < 0.001; *ES* = 0.412) in the presence or absence of rules when comparing LA with and without war connotations. The quasi-sporting games (Play) were mainly warlike activities (*n* = 35; *AR* = 4.2; 34.3%) rather than non-warlike (*n* = 15; *AR* = −4.2; 14.7%). The trend was reversed in relation to games (Game); a greater presence in non-war LA (*n* = 37; *AR* = 4.2; 36.3%) than in war (*n* = 15; *AR* = −4.2; 14.7%) was detected.

### Presence of Motor Interaction in LA With or Without a Warlike Character

Significant differences were found (*X*^2^ = 9.029; *df* = 1; *p* < 0.003; *ES* = 0.298) in the presence or absence of motor interaction when comparing LA with and without war connotations. The psychomotor LA (Psycho) were mainly warlike activities (*n* = 35; *AR* = 3; 34.3%) more than non-warlike (*n* = 21; *AR* = −3; 20.6%). The trend was reversed in relation to sociomotor LA (Socio): greater presence in non-war LA (*n* = 31; *AR* = 3; 30.4%) than war LA (*n* = 15; *AR* = −3; 14.7%).

In addition, there were significant differences in sociomotor LA (*X*^2^ = 28.361; *df* = 3; *p* < 0.001; *ES* = 0.527) when comparing the two groups of LA according to the types of motor interaction: Cooperative LA: non-warlike (*n* = 5; *AR* = −2.2; 4.9%) vs. warlike TSG (*n* = 13; *AR* = 2.2; 12.7%); Opposition LA (Oppo): non-warlike (*n* = 16; *AR* = 4.3; 15.7%) vs. warlike (*n* = 0; *AR* = −4.3; 0%). Cooperation and opposition LA (Coop – opo): non-war LA (*n* = 10; *AR* = 2.4; 9.8%) vs. war LA (*n* = 2; *AR* = −2.4; 2%).

### Presence of Uncertainty in Space in Ludomotor Practices With or Without a Warlike Character

A significant superiority of stable spaces (Stab) over unstable ones (Unstab) was observed (*X*^2^ = 5.056; *df* = 1; *p* < 0.025; *ES* = 0.223), both in warlike LA: Stab (*n* = 50; *AR* = 2.2; 49%) vs. Unstab (*n* = 0; *AR* = −2.2; 0%), and in non-warlike LA: Stab (*n* = 47; *AR* = −2.2; 46.1%) vs. Unstab (*n* = 5; *AR* = 2.2; 4.9%).

### Presence of Materials in LA With or Without a Warlike Character

In both types of LA (with and without war connotations) the presence of materials (Obj) predominated (*X*^2^ = 7.227; *df* = 1; *p* < 0.007; *ES* = 0.266) with respect to LA without objects (No obj). Warlike LA: Obj (*n* = 50; *AR* = 2.7; 49.0%) vs. Non-object (*n* = 0; *AR* = −2.7; 0%); Non- Warlike LA Obj (*n* = 45; *AR* = −2.7; 44.1%) vs. Non-war Obj (*n* = 7; *AR* = 2.7; 6.9%).

### Presence of Accounting in LA With or Without a Warlike Character

There were no statistically significant differences (*X*^2^ = 5.056; *df* = 3; *p* = 0.168; *ES* = 0.222) according to the score by both LA.

### The Age of the Protagonists in LA With or Without a Warlike Character

There were statistically significant differences (*X*^2^ = 5.048; *df* = 3; *p* < 0.001; *ES* = 0.756) when comparing the different ages of the warlike and non-warlike LA. In the wartime LA, mixed age groups predominated: Any age (All): Wartime LA (*n* = 20; *AR* = 3.3; 19.6%) vs. non-wartime LA (*n* = 6; *AR* = −3.3; 5.9%); Children and youth (Child-Youth): Wartime LA (*n* = 30; *AR* = 4.7; 29.4%) vs. non-wartime LA (*n* = 8; *AR* = −4.7; 7.8%). In contrast, the child population only participated in non-warlike LA (*n* = 35; *AR* = 7.2; 34.3%). Only young people were observed practicing LA.

### The Gender of the Protagonists in LA With or Without a Warlike Character

Significant differences were found (*X*^2^ = 32.140; *df* = 3; *p* < 0.001; *ES* = 0.561) between both types of LA. The wartime LA were mainly led by male groups (More): wartime LA (*n* = 41; *AR* = 5.4; 40.2%) vs. non-wartime LA (*n* = 15; *AR* = −5.4; 14.7%). Mixed groups predominated in the non-wartime LA: non-wartime LA (*n* = 34; *AR* = 5.1; 33.3%) vs. wartime LA (*n* = 8; *AR* = −5.1; 7.8%). Hardly any LA were found to be solely male or played by both genders separately.

### The Origin of the Material in LA With or Without a Warlike Character

There were significant differences (*X*^2^ = 10.661; *df* = 3; *p* < 0.014; *ES* = 0.323) between both types of LA. Both groups of LA were mostly made up of objects from the material environment, especially those of a warlike nature: warlike LA (*n* = 50; *AR* = 3.3; 49%) vs. non-warlike LA (*n* = 42; *AR* = −3.3; 41.2%). Scarcely were LA found with objects from a natural environment.

### The Zones in LA With or Without Warlike Character

No significant differences were found (*X*^2^ = 3.722; *df* = 2; *p* = 0.156; *ES* = 0.191) between both types of LA.

### The Calendar in LA With or Without a Warlike Character

All the activities identified were carried out without a calendar.

#### Predictive Capacity of the Ethnomotor Variables of the Internal Logic and the External Logic of LA With or Without a Warlike Character

**FIGURE 1:**
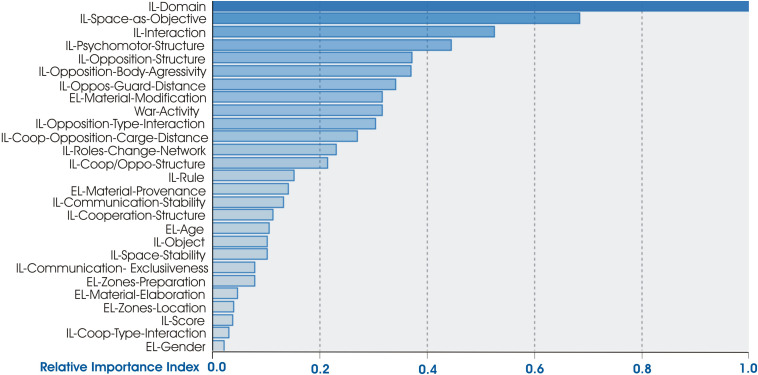
Predictive importance of ethnomotor variables (IL and EL).

The ethnomotor variables were classified into five groups according to their predictive importance (PI), which ranged from 1 (maximum PI) to 0 (low PI) (see Figure 1).

1. The first group (PI = 1) was constituted by the variable IL-Domain (Psycho-Coop-Oppo-Coop/Oppo);
2. The second group (PI ≤ 0.8) was composed of the variable IL-Space-as-objective;
3. The third group (PI ≤ 0.6) was integrated by two variables: IL-Interaction (Psycho-Socio); IL-Psycho-Structures;
4. The fourth group (PI ≤ 0.4) was composed of 13 variables: IL-Opposition-Structures; IL-Opposition-Body-aggression (Oppo-By simple contact; Oppo-Body-contact-objects; Oppo-Strong hit to body; Oppo Permanent body contact; No Oppo body contact); IL-Opposition-Guard-Distance; EL-Material Modification; EL-War-Activity; IL-Opposition Type Interaction; IL-Coop-Opposition-Charge-Distance; IL-Roles-Changes (Fixed, Local, General); IL-Coop/Oppo-Structure;
5. The fifth group (PI ≤ 0.2) was composed of 14 variables: IL-Rule; EL-Material-Provenance; IL-Communication Stability Net; IL-Coop-Structure; EL-Age; IL-Object; ILSpace Stability; IL-Communication Exclusive Net; EL-Zones-Preparation; EL-Material Elaboration; EL-Zones-Location; IL-Score; IL-Coop-Type-Interaction; EL-Gender.

**FIGURE 2:**
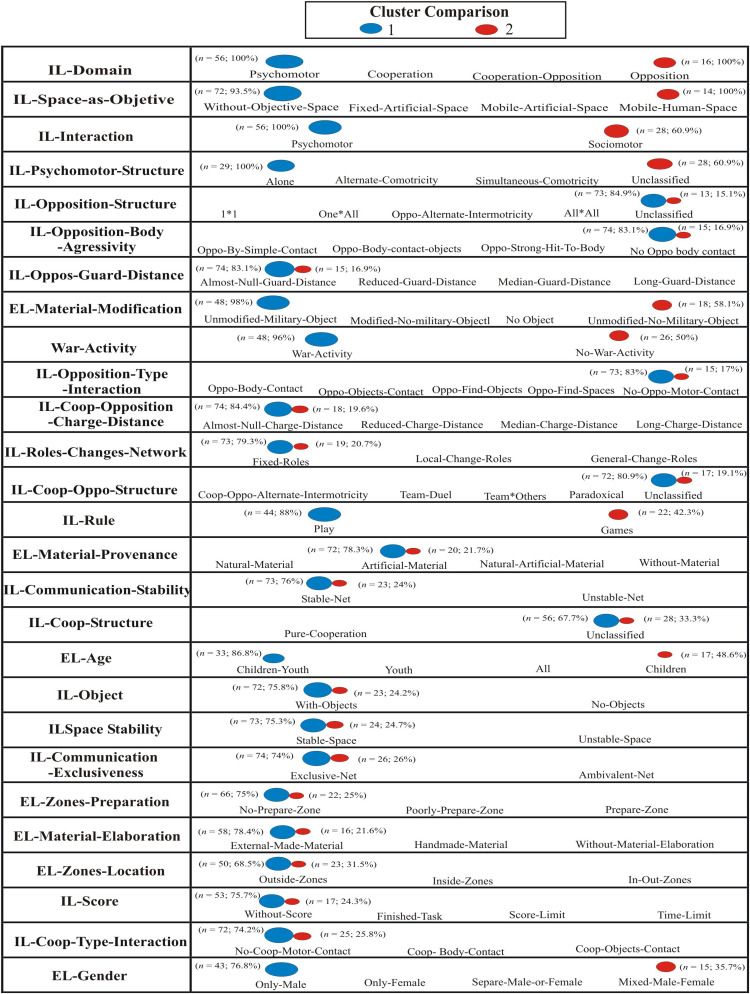
Cluster 1 (Warlike LA) and Cluster 2 (Non- Warlike LA) comparison of the most important ethnomotor categories in each ethnomotor variable.

Figure 2 shows graphically the distribution of the percentages of the predominant category in both clusters 1 (warlike LA) and 2 (non-warlike LA). The first four variables that were of greater predictive capacity in Figure 1 are described below.

The largest cluster (cluster 1) had 68.6% of the cases (*n* = 70) while the smallest one (Cluster 2) had 31.4% (*n* = 32); the ratio of sizes was 2:19. Cluster quality achieved was close to 0.5 and considered “fair” (Schwartz’s Bayesian Criterion (BIC). For interpreting this value, it is necessary to take into account the quality cluster (silhouette measure: both cohesion and separation, ranges from −1 to +1). A negative value in the silhouette measure means that the average distance of a case to members of its own cluster was larger than when compared with other clusters (this is an undesirable feature); however, the results of this study were always positive values in the silhouette measure.

The percentages of the first predictor variable IL domain were unequal in both clusters: Psycho: Cluster 1 (*n* = 56; 100%) vs. Cluster 2 (*n* = 0; 0%); Coop: Cluster 1 (*n* = 18; 100%) vs. Cluster 2 (*n* = 0; 0%); Oppo: Cluster 1 (*n* = 0; 0%); vs. Cluster 2 (*n* = 16; 100%); Coop-Oppo: Cluster 1 (*n* = 0; 0%); vs. Cluster 2 (*n* = 12; 100%).

The second predictive variable IL-Space as objective originated unequal percentages in both clusters: Without Objective space: Cluster 1 (*n* = 72; 93.5%) vs. Cluster 2 (*n* = 5; 6.5%); Fixed artificial space: Cluster 1 (*n* = 2; 22.2%) vs. Cluster 2 (*n* = 7; 77.8%); Mobile artificial space: Cluster 1 (*n* = 0; 0%); vs. Cluster 2 (*n* = 2; 22.2%); Mobile human space: Cluster 1 (*n* = 0; 0%); vs. Cluster 2 (*n* = 14; 100%).

The percentages of the third variable IL-Interaction were unequal in both clusters: Psychomotor: Cluster 1 (*n* = 56; 100%) vs. Cluster 2 (*n* = 0; 0%); Sociomotor: Cluster 1 (*n* = 18; 39.1%) vs. Cluster 2 (*n* = 28; 60.9%).

The fourth variable IL-Psycho-Structures also generated different percentages in both clusters: Alone cluster 1 (*n* = 29; 100%) vs. Cluster 2 (*n* = 0; 0%); Altern Comotricity cluster 1 (*n* = 16; 100%) vs. Cluster 2 (*n* = 0; 0%); Simultaneous Comotricity cluster 1 (*n* = 11; 39.1%) vs. Cluster 2 (*n* = 0; 0%); Not Psychomotor structures Cluster 1 (*n* = 18; 100%) vs. Cluster 2 (*n* = 28; 60.9%).

The variable war or non-warlike LA established a clear distinction between both families of LA: Cluster 1 (*n* = 48; 96.0%) vs. Cluster 2 (*n* = 2; 4.0%); No-warlike LA: Cluster 1 (*n* = 2; 4%); vs. Cluster 2 (*n* = 26; 50%).

## Discussion

This research studied the distinctive ethnomotor features of the ludomotor activities (LA) with or without rules carried out in the city of Lleida (Catalonia, Spain) in the context of the Spanish Civil War (1936–1939). Likewise, the features of LA with and without war connotations were compared.

This study presents an original approach to statistical analysis by means of descriptive statistics, Crosstab’s Command, and clusters of the 27 ethnomotor variables of the identified LA. These statistical strategies allowed revealing and interpreting the ethnomotor features of LA with and without war connotations as interlaced units of a unitary set. In this way, the following findings are highlighted.

## Ludomotor Activities in the Context of the War Contain Unique Ethnomotor Feature

The unitary interpretation of the four ethnomotor variables with the greatest predictive force allows us to understand the uniqueness of the LA studied in the context of the Spanish Civil War [predictive variables: (a) domain of motor action; (b) space as an objective to be reached, (c) sociomotor or psychomotor; and (d) structures of the psychomotor domain].

This systemic ethnomotor vision justifies that the body of techniques (Mauss, 1996) introduced by the protagonists can be categorized into two large groups of ludomotor experiences:

(a) Psychomotricity, constituted by individual LA with the participation of the players, was carried out without interaction with other people. The participants had a variety of options, that is, using psychomotor structures to test their autonomy and exploratory capacity. This form of LA involved playing alone (solitary) or participating with others in turns, without helping or hurting each other (alternative comotricity) or in separate spaces, or without being able to interact with others (simultaneous comotricity). Psychomotor LA accounted for almost half of all LA, a percentage much higher than that found in other periods without military conflict (Lavega et al., 2006). To interpret this regularity, it will be necessary to go further into the distinctive ethnomotor features of LA with war connotations. This regularity has never been observed in other types of LA in Catalonia (Lavega et al., 2006) or cultural contexts in Europe (e.g., Etxebeste, 2001; Lavega et al., 2006; Navarro-Adelantado and Lavega, 2017; Lavega-Burgués and Navarro-Adelantado, 2019).

(b) Sociomotricity, represented by LA where players can share motor interaction with others. We observed the prevalence of LA with opponents that served to socialize interpersonal relationships. The bodies of the opponents become a target to be reached, that tests the decision making of the participants (Lavega-Burgués et al., 2020). This regularity is also observed in other moments in LA in Catalonia (Lavega et al., 2006). In order to interpret this group of LA, it will be convenient to go deeper into the features of the LA without warlike features.

## The Ethnomotor Feature of LA With and Without he Warlike Connotations

Statistical analyses identified unique ethnomotor traits for LA with and without war connotations. Therefore, ethnomotor interpretation should consider the regularities of these two families of LA in the context studied (Ormo, 2017).

In both cases, it will be highlighted in which way the internal logic of these practices activates the subjectification of the participants (Warnier, 2011). The intention is to reveal the power offered by LA to achieve autonomy over their own body of techniques and over the understanding of the network of values shared with the other players.

### LA With War Connotations as Agents of Material Subjectification

Statistical analyses revealed that one of the main distinctive features of the LA was the ludic use of war materials (bombs, grenades, etc.). Younger men (boys and young men) used quasi-games (QTSG) to explore the cold, serious, and dangerous weaponry used by the military. Both in war and in ludic activities, the final objective is to make these materials explode, in the first case on the enemies, in the second, on a human target. Thus, these activities were QTSG, i.e., activities without rules, open to constant changes in game actions, at the will of the participants. They were mostly psychomotor (Figure 2, Cluster 1), which is in line with previous research (Alonso et al., 2010) but against the findings in the Olympic Games (Parlebas, 1988; Pic, 2018; Parlebas, 2020), and absent of any motor interaction with others, that is, they allowed testing the self-sufficiency of each person in risky situations. Exploration, creativity, fantasy, and overcoming of fear were constant in the motor behaviors associated with the process of subjectification of the material culture of this moment. Thanks to these body of techniques, the players educated themselves on the competencies of autonomy and personal initiative (European Union., 2006; European Commission/Eacea/Eurydice, 2012) in situations of maximum risk (Warnier, 2002).

### LA Without War Connotations as Agents of Social Subjectification

LA without war connotations were mostly games (TSG), that is, activities with traditional rules that had already been practiced by previous generations (Lavega et al., 2006). They were sociomotor TSG based on motor interactions with others. The players (children who participated in mixed male and female groups) learned to interact and enjoy the group encounter with others (Parlebas, 2003). These activities were mainly of an oppositional nature, consisting of looking for the body of others that became a mobile target to be reached, as in the family of TSG represented by persecution (such as the one against all or all against all structure in tag games). When the TSG used some materials, the objects came from the environment and were not modified, that is, they were used in the conditions in which they were found. Examples are usually games where the rivalry is established through the contact of the other objects (e.g., marble games) (Etxebeste, 2001). This kind of body of techniques helped to educate the process of social subjectification, that is, to understand and internalize the signs, messages, and interpersonal values important in that society (Berthoud-Aghilin, 1986; Warnier, 2011).

## Limitations

This study had a chronological limitation. The population able to be interviewed was limited to people over 69 years old at the time of the interviews. In addition, they had to be residents in the area at the time of the events, willing to express their personal experiences, and with the intellectual capacity to remember and maintain the conversation during the interview.

## Future Perspective

It would be interesting to carry out this same study in other war contexts (in other countries) to identify ethnomotor traits and compare them with those found in the studied environment.

## Conclusion

This study confirms the need for contextualized research (in a specific place and historical moment) to interpret LA (with rules/TSG or without rules/QTSG) in relation to local cultures. LA show unique ethnomotor features due to the presence of war.

This study successfully addressed the contextualized identification and cultural interpretation of the ethnomotor features of LA practiced during the Spanish civil war.

The use of the theoretical fundamentals of the science of motor action, together with the variety of statistical analyses used, have enabled us to reveal the main ethnomotor properties of LA described by the people interviewed.

The organization of the predictive capacity of the variables suggests the predominant importance of the domain (type of motor relationship in LA). This identification was crystallized in a more concrete motor silhouette of individual participation and warlike reminiscence (Cluster 1), as opposed to sociomotor participation and non-warlike character (Cluster 2). This study confirms the need to conduct further ethnomotor research to reveal the connection of LA in war contexts. Furthermore, thanks to ethnomotricity, it is easier to understand the process of subjectification that LA creates.

## Data Availability Statement

The raw data supporting the conclusions of this article will be made available by the authors, without undue reservation.

## Ethics Statement

The studies involving human participants were reviewed and approved by the ethics committee for clinical research of the catalan sports council. The patients/participants provided their written informed consent to participate in this study.

## Author Contributions

EO-R, PL-B, and MP: substantial contribution to study conception and design. EO-R, PL-B, RR-A, and AR-A: preparation of the document for approval by the ethics committee. EO-R, PL-B, and RL-P: preparation and participation in the empirical work. EO-R, PL-B, RR-A, RL-P, AR-A, and MP: database revision. EO-R, PL-B, RL-P, and MP: discussion of data analysis strategies. EO-R, PL-B, RR-A, RL-P, AR-A, and MP: writing of the manuscript. All authors contributed to the article and approved the submitted version.

## Conflict of Interest

The authors declare that the research was conducted in the absence of any commercial or financial relationships that could be construed as a potential conflict of interest.

## Footnotes

1 http://praxiologiamotriz.inefc.es/?p=864
